# Diagnostic Performance of CSF Interleukin-10 in Primary Central Nervous System Lymphoma: A Retrospective Study

**DOI:** 10.7759/cureus.89063

**Published:** 2025-07-30

**Authors:** Eliezer Villanueva-Castro, José Guillermo Flores-Vázquez, Rebeca Hernández Reséndiz, Luis A Rodríguez-Hernández, Ivan Abdiel Rodríguez-Hernández, Rodolfo Villalobos-Díaz, Tomas Moncada-Habib, Marco Antonio Muñuzuri-Camacho, Edgardo de Jesús Mateo-Nouel, Xavier Wong-Achi, Ricardo Alberto Palacios-Rodríguez, Bernardo Cacho-Díaz, Guillermo Axayacalt Gutierrez-Aceves, Sergio Moreno-Jiménez, Alberto González-Aguilar

**Affiliations:** 1 Neurosurgery, Instituto Nacional de Neurología y Neurocirugía Manuel Velasco Suárez, Mexico City, MEX; 2 Neurology, Hospital Angeles Universidad, Mexico City, MEX; 3 Mycology, Hospital General Dr. Manuel Gea González, Mexico City, MEX; 4 Neuro-Oncology, Instituto Nacional de Cancerología, Mexico City, MEX; 5 Neurosurgery, Instituto Nacional de Neurología y Neurocirugía Manuel Velasco Suárez, México City, MEX; 6 Neurosurgery, Centro Médico American British Cowdray, Mexico City, MEX

**Keywords:** biomarker, brain tumor, cerebrospinal fluid, interleukin-10, primary central nervous system lymphoma

## Abstract

Background: Primary central nervous system lymphoma (PCNSL) remains a diagnostic challenge due to its radiological overlap with other brain lesions and limitations of stereotactic biopsy, particularly following corticosteroid exposure. Interleukin-10 (IL-10) is a cytokine frequently elevated in PCNSL and has emerged as a potential diagnostic biomarker, yet reported cut-off values vary widely, limiting clinical application.

Objective: To determine the diagnostic accuracy of CSF IL-10 for differentiating PCNSL from other brain lesions by estimating the optimal cut-off, sensitivity, specificity, and area under the receiver operating characteristic (ROC) curve (AUC), and to explore its potential prognostic value.

Materials and methods: We conducted a retrospective observational study of 115 patients who underwent lumbar puncture for diagnostic workup of brain lesions at a tertiary referral center between 2015 and 2020. CSF IL-10 levels were measured using a standardized enzyme-linked immunosorbent assay (ELISA) protocol. Patients with prior corticosteroid use, HIV infection, insufficient CSF, or lacking a definitive diagnosis were excluded. Diagnostic performance was assessed via ROC analysis. The association between CSF IL-10 and progression-free survival (PFS) was explored in patients with histologically confirmed PCNSL receiving standard therapy.

Results: Sixty-three patients were diagnosed with PCNSL, and 52 with alternative pathologies, including gliomas, demyelinating diseases, and infections. The mean CSF IL-10 level was significantly higher in PCNSL patients (109.9 pg/mL) compared to non-PCNSL (12.6 pg/mL). Using a cut-off of 20.05 pg/mL, CSF IL-10 showed a sensitivity of 93.7% (95% CI: 84.8-97.5%) and specificity of 88.5% (95% CI: 77.0-94.6%), with an AUC of 0.95 (95% CI: 0.91-0.99). The positive predictive value was 90.8% and the negative predictive value was 92.0%. Higher IL-10 levels were modestly correlated with shorter PFS (R^2^ = 0.315, p < 0.015).

Conclusions: CSF IL-10 quantification may serve as a minimally invasive, high-yield adjunct in the diagnosis of PCNSL, particularly when biopsy is delayed or contraindicated. However, as both the diagnostic cut-off and prognostic correlation were derived from the same retrospective cohort, prospective validation is essential prior to clinical adoption.

## Introduction

Primary central nervous system lymphoma (PCNSL) is an aggressive extranodal non-Hodgkin lymphoma that accounts for 1-6% of all central nervous system malignancies. Its clinical and radiological presentations often overlap with other intracranial pathologies such as high-grade gliomas, metastases, demyelinating diseases, and infections, complicating diagnosis [1-7].

Although neuroimaging typically reveals homogeneously enhancing periventricular lesions, these findings lack specificity [8-11]. Stereotactic biopsy remains the diagnostic gold standard; however, its accuracy can be compromised by corticosteroid exposure, which may reduce lesion visibility or cause transient remission. Additionally, sampling errors and limited tissue yield may lead to misdiagnosis, with error rates reported as high as 30% [12-15].

Cerebrospinal fluid (CSF) biomarkers, particularly interleukin-10 (IL-10), have emerged as promising adjuncts in the diagnostic workup of PCNSL. IL-10 is a cytokine commonly elevated in lymphoproliferative disorders, and its quantification in CSF has demonstrated high sensitivity and specificity in prior studies [7,9-11,13,14,16]. Although multiple studies support the diagnostic value of IL-10, the reported cut-off values vary widely, from 2 to 21.7 pg/mL, depending on assay type, cohort characteristics, and inclusion criteria. These discrepancies hinder standardization and limit generalizability. Our study contributes to the existing literature by proposing a high-specificity threshold in a rigorously selected cohort, excluding pre-treated patients and encompassing a broad differential diagnosis. In this retrospective cohort, we sought to establish the diagnostic accuracy of CSF IL-10 for differentiating PCNSL from other brain lesions by estimating the optimal cut-off, sensitivity, specificity, and area under the receiver operating characteristic (ROC) curve (AUC).

## Materials and methods

This study is a single-center, retrospective observational analysis conducted at the Instituto Nacional de Neurología y Neurocirugía Manuel Velasco Suárez, Mexico City, Mexico. We included consecutive patients with brain lesions who underwent diagnostic lumbar puncture and CSF IL-10 quantification as part of their routine diagnostic workup between 2015 and 2020. All data were obtained from existing medical records and laboratory databases.

Sample collection and laboratory processing

Pre-analytical handling of CSF samples followed standardized institutional protocols. A minimum of 2 mL of CSF was collected via standard lumbar puncture prior to any treatment initiation. Samples were processed within one hour of collection, centrifuged at 1500 g for 10 minutes, and stored at -80°C until analysis. IL-10 quantification was performed using Human IL-10 Quantikine® ELISA kits (R&D Systems, Minneapolis, MN, USA; catalog #D1000B) according to the manufacturer’s instructions. All samples were run in duplicate. The intra-assay and inter-assay coefficients of variation were 4.2% and 6.7%, respectively. Laboratory personnel were not blinded to clinical diagnoses, which may introduce measurement bias.

Eligibility criteria and diagnostic definitions

The study included all CSF sampling of patients with brain lesions such as primary brain tumors, demyelinating diseases, metastases, and neuroinfectious diseases. PCNSL was confirmed by stereotactic or open brain biopsy in all cases. For non-PCNSL diagnoses, gliomas were confirmed by histopathology following surgical resection or biopsy; demyelinating diseases were diagnosed based on characteristic MRI features and CSF analysis (e.g., oligoclonal bands or elevated IgG index); infectious etiologies were diagnosed via CSF microbiological culture, polymerase chain reaction (PCR), or antigen detection. No additional procedures or interventions were performed beyond routine clinical care.

Patients were excluded if they met any of the following criteria: (1) prior corticosteroid use before CSF collection, (2) insufficient CSF volume for IL-10 quantification, (3) lack of a definitive diagnosis, or (4) HIV infection. The exclusion of HIV-positive patients was based on the known immunomodulatory effects of HIV, which can increase IL-10 levels and reduce the diagnostic specificity of this biomarker in HIV-related lymphomas.

Cohort description and patient flow

**Figure 1:**
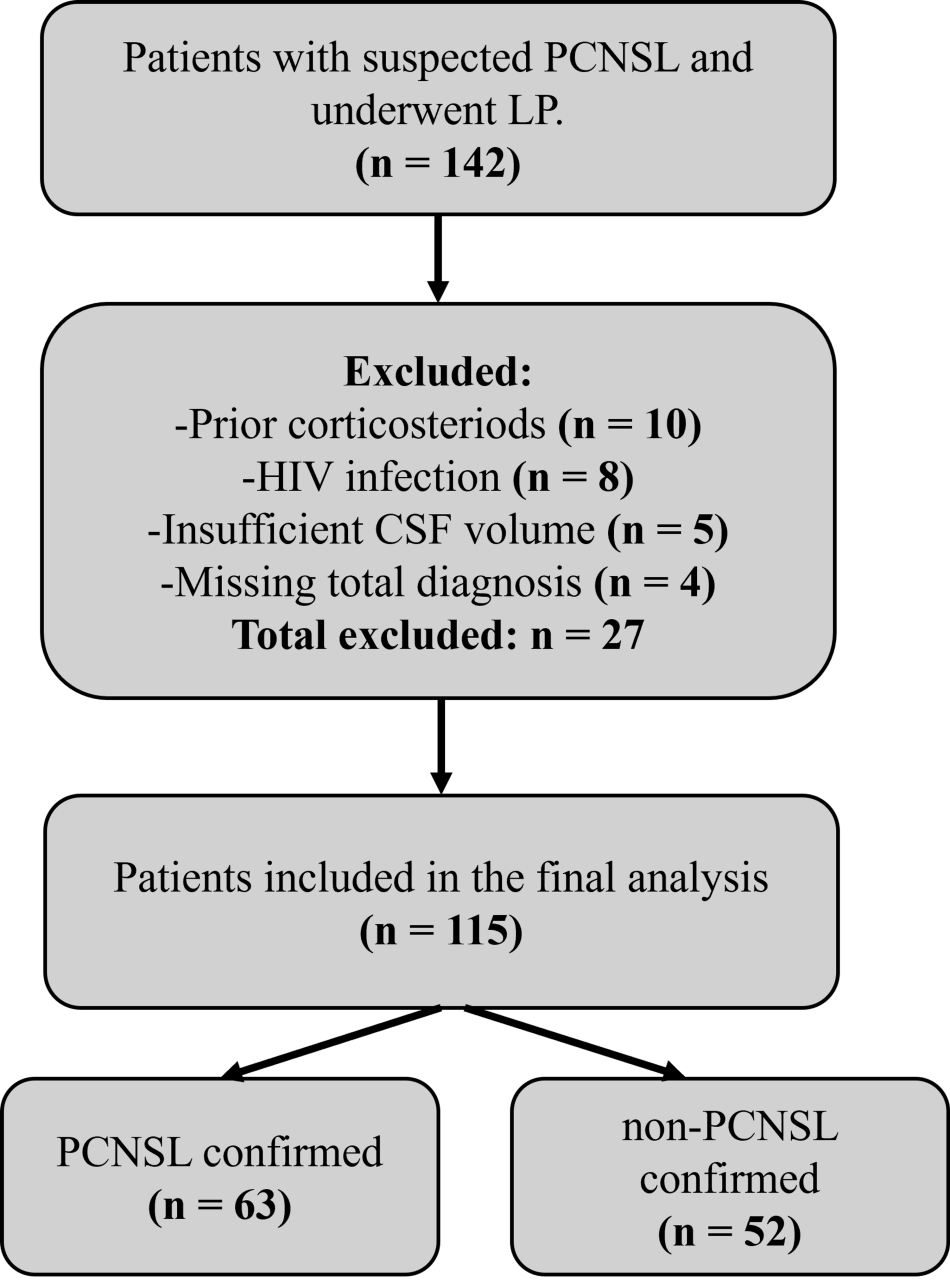
Patient flow diagram illustrating cohort selection A total of 142 patients underwent diagnostic lumbar puncture for evaluation of suspected brain lesions between 2015 and 2020. Twenty-seven patients were excluded due to prior corticosteroid use (n = 10), HIV infection (n = 8), insufficient cerebrospinal fluid (CSF) volume (n = 5), or lack of a definitive diagnosis (n = 4). The final analysis included 115 patients, of whom 63 were diagnosed with primary central nervous system lymphoma (PCNSL) and 52 with non-PCNSL conditions (e.g., gliomas, demyelinating diseases, infections, etc.).

A total of 142 patients underwent diagnostic lumbar puncture for suspected brain lesions during the study period. Twenty-seven were excluded for the following reasons: prior corticosteroid use (n = 10), HIV infection (n = 8), insufficient CSF volume for IL-10 analysis (n = 5), and lack of a definitive diagnosis (n = 4). The final cohort included 115 patients (Figure 1).

Statistical analysis

Normality was assessed using the Shapiro-Wilk test, and variance homogeneity with Levene’s test. IL-10 levels between PCNSL and non-PCNSL groups were compared using the non-parametric Mann-Whitney U test. ROC curve analysis was used to estimate the diagnostic performance of CSF IL-10 and determine the optimal cut-off. AUC significance was defined as different from 0.5, and 95% confidence intervals (CIs) were reported.

Progression-free survival (PFS) was defined as the time from lumbar puncture to radiographic progression or death. Survival analyses included only patients with histologically confirmed PCNSL who received standard treatment (high-dose methotrexate-based chemotherapy with or without radiotherapy). Correlation between CSF IL-10 levels and PFS was assessed using Pearson's correlation coefficient.

A post-hoc power analysis was conducted using observed means, group sizes, and a standard deviation of 25 pg/mL. With 63 PCNSL and 52 non-PCNSL cases, an effect size of Cohen’s d = 0.54 was observed, yielding a statistical power of 81.4% at α = 0.05. All analyses were performed using IBM SPSS Statistics for Windows, Version 20 (Released 2012; IBM Corp., Armonk, New York, United States), with statistical significance set at p < 0.05.

## Results

A total of 115 patients were enrolled in the study, with a mean age of 51.4 years. Of these, 51 (44%) were women and 64 (56%) men. The cohort was divided into two groups: 63 patients (54.8%) with PCNSL and 52 patients (45.2%) with non-PCNSL diagnoses. Among the non-PCNSL group, 36 (69%) had brain tumors (e.g., gliomas, metastases), five (9.6%) had neuroinfectious diseases, five (9.6%) had inflammatory demyelinating disorders, and six (11.5%) had other conditions.

The mean CSF protein level in the full cohort was 64.4 mg/dL. Hypoglycorrhachia (CSF glucose <40 mg/dL) was observed in 22 of 115 patients (19.1%), and CSF pleocytosis (>5 cells/µL) in 39 patients (33.9%). The mean CSF cell count was 17.5 cells/µl.

Median CSF IL-10 levels were significantly higher in the PCNSL group (109.9 pg/mL) compared to the non-PCNSL group (12.6 pg/mL) (p < 0.001) (Table 1).

**Table 1:** PCNSL and non-PCNSL groups characteristics PCNSL: primary central nervous system lymphoma; CFS: cerebrospinal fluid; IL-10: interleukin-10

Demographic variables	PCNSL	Non-PCNSL
Total patients (n)	63	52
Age (years) (mean)	51.5	51
Sex (male) (n)	37	27
Sex (female) (n)	26	25
CFS proteins (mg/dL) (mean)	76.153	49.61
CFS IL-10 levels (pg/dL) (mean)	109.94	12.58

**Figure 2:**
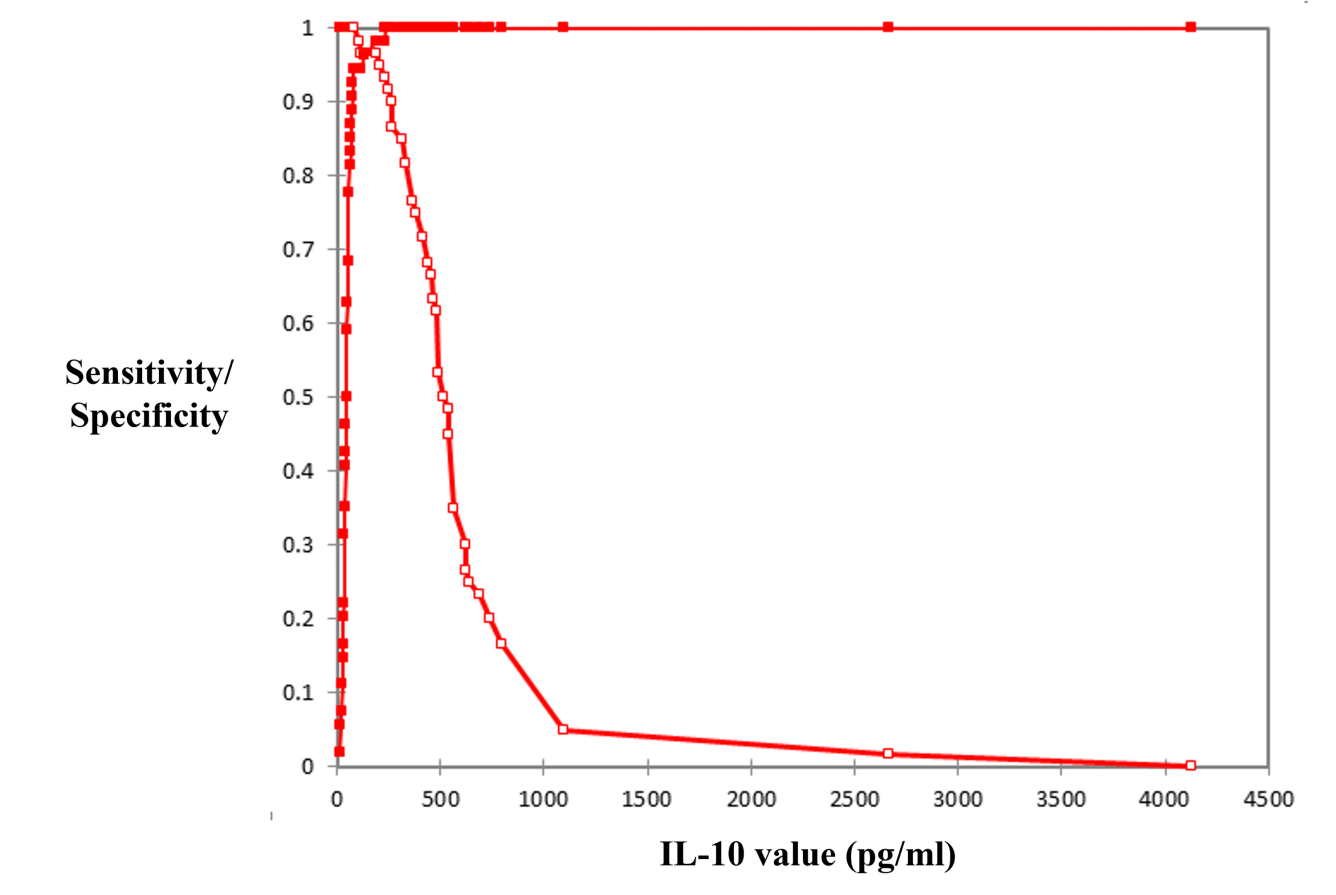
Receiver operating characteristic (ROC) curve of CSF IL-10 for PCNSL diagnosis ROC analysis demonstrating the diagnostic performance of cerebrospinal fluid (CSF) IL-10 levels for distinguishing PCNSL from other brain lesions. A cut-off value of 20.05 pg/mL yielded an area under the curve (AUC) of 0.95 (95% CI: 0.91-0.99), with a sensitivity of 93.7% and specificity of 88.5%. PCNSL: primary central nervous system lymphoma; IL-10: interleukin-10

Using a cut-off value of 20.05 pg/mL, CSF IL-10 demonstrated a sensitivity of 93.7% (95% CI: 84.8-97.5%) and specificity of 88.5% (95% CI: 77.0-94.6%) for diagnosing PCNSL. The positive predictive value (PPV) was 90.8% (95% CI: 81.3-95.7%), and the negative predictive value (NPV) was 92.0% (95% CI: 81.2-96.8%). The likelihood ratios were LR(+) = 8.12 (95% CI: 3.81-17.27) and LR(-) = 0.072 (95% CI: 0.028-0.186), indicating strong discriminatory performance of CSF IL-10 for distinguishing PCNSL from other intracranial pathologies (Figure 2).

**Figure 3:**
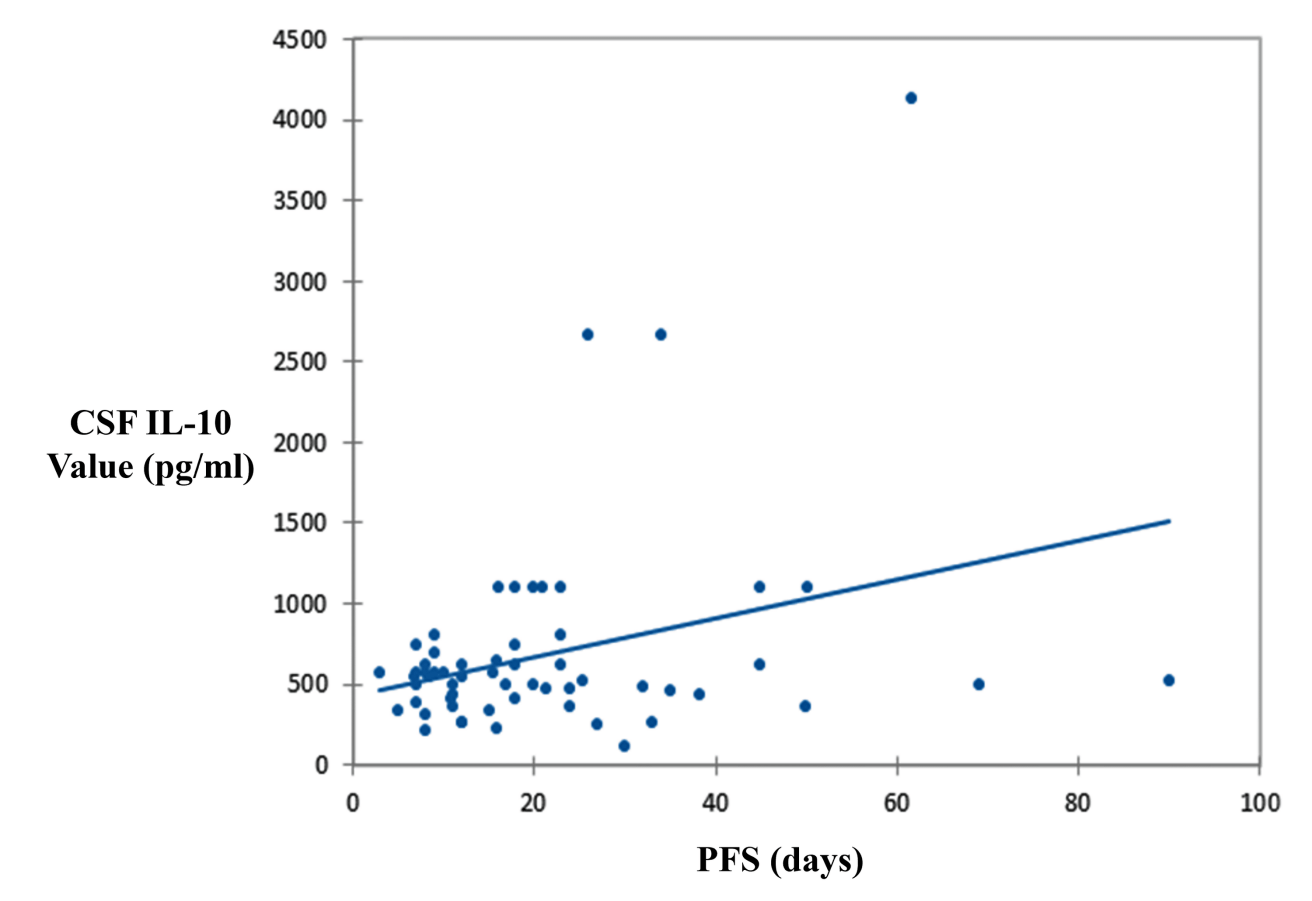
Correlation between baseline CSF IL-10 levels and PFS in PCNSL patients Scatterplot illustrating the relationship between CSF IL-10 concentration at diagnosis and progression-free survival (PFS) in patients with histologically confirmed PCNSL who received standard treatment. A modest inverse correlation was observed (R^2^ = 0.315, p < 0.015), suggesting that higher IL-10 levels may be associated with shorter PFS. PCNSL: primary central nervous system lymphoma; CFS: cerebrospinal fluid; IL-10: interleukin-10

Additionally, a subgroup of 50 PCNSL patients who received standard therapy was included in a PFS analysis. A modest but statistically significant negative correlation was observed between baseline CSF IL-10 levels and PFS (Pearson’s r = -0.56, R^2^ = 0.315, p = 0.014), suggesting that higher IL-10 levels may be associated with shorter PFS. The strength of this association is modest. However, this exploratory finding requires prospective validation and multivariable adjustment to account for potential confounding (Figure 3).

## Discussion

PCNSL has shown a rising incidence in recent decades. Although neuroimaging often reveals enhancing periventricular lesions, these findings are not pathognomonic and frequently overlap with other neoplastic and non-neoplastic conditions such as gliomas, metastases, demyelinating diseases, and infections [8,17-24]. Despite technological advances, histopathological confirmation remains the diagnostic cornerstone.

Stereotactic biopsy is considered the gold standard but may yield false negatives due to corticosteroid-induced tumor regression, sampling variability, or limited tissue yield. Misdiagnosis rates have been reported in up to 30% of cases [19,20]. Moreover, PCNSL displays marked radiological heterogeneity, with at least 18 described MRI patterns, complicating its differentiation from other intracranial entities [25-27].

Given these limitations, CSF biomarkers such as IL-10 have emerged as promising adjuncts to support early diagnosis, particularly when biopsy is delayed, contraindicated, or inconclusive. However, they are not intended to replace tissue confirmation. Our study contributes to this growing body of evidence by evaluating the diagnostic and exploratory prognostic utility of CSF IL-10 in a rigorously selected, steroid-naïve cohort with broad differential diagnoses.

Elevated CSF IL-10 concentrations were observed in the majority of patients with PCNSL. This increase is believed to result from aberrant IL-10 secretion by malignant B cells, which fosters immune evasion by suppressing antigen presentation, inhibiting pro-inflammatory cytokines, and promoting regulatory T-cell activity. These immunosuppressive mechanisms promote tumor persistence and progression.

At a cut-off of 20.05 pg/mL, CSF IL-10 demonstrated high diagnostic accuracy (sensitivity 93.7%, specificity 88.5%), consistent with previous studies despite the variability in reported thresholds (ranging from 2 to 21.7 pg/mL) and test performance across different platforms and populations (Table 2) [15-17,28-32]. Our results may reflect enhanced internal validity conferred by standardized protocols and exclusion of corticosteroid-pretreated patients, a factor known to affect cytokine profiles.

**Table 2:** Comparison of diagnostic thresholds and performance metrics of CSF IL-10 across selected studies Summary of key findings from previous studies evaluating CSF IL-10 as a diagnostic biomarker for PCNSL. Includes reported cut-off values, sample sizes, sensitivity, specificity, and assay details when available. Note the variability in diagnostic thresholds (ranging from 2 to 21.7 pg/mL) and performance metrics, which may reflect differences in assay platforms, patient selection criteria, and inclusion of corticosteroid-pretreated individuals. The present study is highlighted in bold for reference. PCNSL: primary central nervous system lymphoma; CFS: cerebrospinal fluid; IL-10: interleukin-10; ADC: apparent diffusion coefficient

Author	Year	Cut-off (pg/mL)	Sensitivity (%)	Specificity (%)	Observations	Sample size (n)
Sasayama et al. [15]	2012	9.5	71	100	Moderate sensitivity, excellent specificity in immunocompetent patients.	26 PCNSL, 40 non-PCNSL
Rubenstein et al. [17]	2013	16.2	64	94.1	Combined with CXCL13 to enhance diagnostic accuracy. Included primary, secondary, newly diagnosed, and recurrent CNSL.	83 CNSL, 137 non-PCNSL
Sasagawa et al. [29]	2015	3	94.7	100	Excellent diagnostic power, small cohort.	19 CNSL and 26 non-lymphoma
Nguyen-Them et al. [28]	2016	4	88.6	88.9	Prospective study with prognostic implications.	79 CNSL and 40 control
Mabray et al. [30]	2016	21.7	62.7	95.4	Compared IL-10 with ADC and CXCL13.	43 PCNSL and 44 control
Song et al. [31]	2016	8.2	95.5	96.1	High sensitivity and specificity using the IL-10/IL-6 ratio.	22 PCNSL and 80 control
Shao et al. [32]	2020	8.3	59	98	Focus on immunocompetent patients.	66 PCNSL and 42 control
Ferreri et al. [16]	2021	2	88	99	Combined with MYD88 mutation detection.	93 PCNSL and 162 control
González-Aguilar et al.	2025	20.05	93	89	Current study: strict exclusion of steroid-pretreated patients.	63 PCNSL and 52 non-PCNSL

Importantly, we found a modest but significant negative correlation between IL-10 levels and PFS (R^2^ = 0.315, p = 0.014). While this suggests moderate prognostic relevance, the finding should be interpreted cautiously given its exploratory nature and lack of adjustment for censoring or clinical confounders. Further prospective studies using multivariable survival analysis are needed to evaluate whether IL-10 independently predicts outcomes in PCNSL.

Several limitations must be acknowledged. First, although the cohort is one of the largest single-center series to date, it remains retrospective and lacks internal or external validation. The diagnostic threshold was derived and tested within the same cohort, potentially inflating diagnostic estimates due to optimism bias. Second, no blinding was applied during laboratory analysis, introducing potential measurement bias. Third, although histological confirmation was obtained in all PCNSL cases, some non-PCNSL diagnoses were based on clinical-radiologic criteria, which may limit diagnostic precision. Finally, we did not explore alternative biomarkers or assess cost-effectiveness, both of which are essential for future implementation. Larger multicenter prospective cohorts are needed to validate the proposed cut-off threshold and confirm reproducibility.

Despite these limitations, our findings support the potential role of CSF IL-10 as an adjunct diagnostic biomarker for PCNSL, especially when biopsy is delayed, contraindicated, or yields inconclusive results. Its minimally invasive nature may be particularly valuable in fragile patients or those with lesions in eloquent or surgically inaccessible regions. Nonetheless, prospective validation is essential before widespread clinical adoption or broad integration into clinical workflows. Future studies should aim to confirm the proposed threshold across diverse populations, harmonize assay procedures, and evaluate cost-effectiveness prior to adoption in routine clinical settings.

## Conclusions

Quantification of IL-10 in CSF shows promise as a minimally invasive, high-yield adjunct in the diagnostic evaluation of suspected PCNSL, particularly when biopsy is delayed, contraindicated, or inconclusive. While our findings support its potential diagnostic utility and suggest a modest correlation with PFS, these results remain exploratory and should be interpreted with caution. Prospective, multicenter studies are necessary to validate the proposed diagnostic threshold, ensure reproducibility across platforms, and determine its independent prognostic value prior to routine clinical integration.

The incorporation of IL-10 measurement into diagnostic workflows may improve early diagnostic accuracy and enable more timely treatment decisions. Its application may be especially valuable in resource-limited settings or when invasive procedures are not feasible. As our understanding of cytokine dynamics in CNS malignancies continues to evolve, biomarkers such as IL-10 may contribute to more precise and personalized diagnostic strategies.

## References

[1] Olson JE, Janney CA, Rao RD (2002). The continuing increase in the incidence of primary central nervous system non-Hodgkin lymphoma: a surveillance, epidemiology, and end results analysis. Cancer.

[2] Pels H, Juergens A, Schirgens I (2010). Early complete response during chemotherapy predicts favorable outcome in patients with primary CNS lymphoma. Neuro Oncol.

[3] Chukwueke U, Grommes C, Nayak L (2022). Primary central nervous system lymphomas. Hematol Oncol Clin North Am.

[4] Kosaka N, Tsuchida T, Uematsu H, Kimura H, Okazawa H, Itoh H (2008). 18F-FDG PET of common enhancing malignant brain tumors. AJR Am J Roentgenol.

[5] Yamashita K, Yoshiura T, Hiwatashi A (2013). Differentiating primary CNS lymphoma from glioblastoma multiforme: assessment using arterial spin labeling, diffusion-weighted imaging, and ¹⁸F-fluorodeoxyglucose positron emission tomography. Neuroradiology.

[6] Scott BJ, Douglas VC, Tihan T, Rubenstein JL, Josephson SA (2013). A systematic approach to the diagnosis of suspected central nervous system lymphoma. JAMA Neurol.

[7] Cassoux N, Giron A, Bodaghi B (2007). IL-10 measurement in aqueous humor for screening patients with suspicion of primary intraocular lymphoma. Invest Ophthalmol Vis Sci.

[8] Lin X, Khan IR, Seet YH, Lee HY, Yu WY (2020). Atypical radiological findings of primary central nervous system lymphoma. Neuroradiology.

[9] Cassoux N, Merle-Beral H, Leblond V (2000). Ocular and central nervous system lymphoma: clinical features and diagnosis. Ocul Immunol Inflamm.

[10] Kimura K, Usui Y, Goto H (2012). Clinical features and diagnostic significance of the intraocular fluid of 217 patients with intraocular lymphoma. Jpn J Ophthalmol.

[11] Wolf LA, Reed GF, Buggage RR, Nussenblatt RB, Chan CC (2003). Vitreous cytokine levels. Ophthalmology.

[12] Correia CE, Schaff LR, Grommes C (2020). Central nervous system lymphoma: approach to diagnosis and treatment. Cancer J.

[13] Chan CC, Buggage RR, Nussenblatt RB (2002). Intraocular lymphoma. Curr Opin Ophthalmol.

[14] Chan CC (2003). Molecular pathology of primary intraocular lymphoma. Trans Am Ophthalmol Soc.

[15] Sasayama T, Nakamizo S, Nishihara M (2012). Cerebrospinal fluid interleukin-10 is a potentially useful biomarker in immunocompetent primary central nervous system lymphoma (PCNSL). Neuro Oncol.

[16] Ferreri AJ, Calimeri T, Lopedote P (2021). MYD88 L265P mutation and interleukin-10 detection in cerebrospinal fluid are highly specific discriminating markers in patients with primary central nervous system lymphoma: results from a prospective study. Br J Haematol.

[17] Rubenstein JL, Wong VS, Kadoch C (2013). CXCL13 plus interleukin 10 is highly specific for the diagnosis of CNS lymphoma. Blood.

[18] Law M, Yang S, Babb JS, Knopp EA, Golfinos JG, Zagzag D, Johnson G (2004). Comparison of cerebral blood volume and vascular permeability from dynamic susceptibility contrast-enhanced perfusion MR imaging with glioma grade. AJNR Am J Neuroradiol.

[19] Coulon A, Lafitte F, Hoang-Xuan K, Martin-Duverneuil N, Mokhtari K, Blustajn J, Chiras J (2002). Radiographic findings in 37 cases of primary CNS lymphoma in immunocompetent patients. Eur Radiol.

[20] Bühring U, Herrlinger U, Krings T, Thiex R, Weller M, Küker W (2001). MRI features of primary central nervous system lymphomas at presentation. Neurology.

[21] Porter AB, Giannini C, Kaufmann T (2008). Primary central nervous system lymphoma can be histologically diagnosed after previous corticosteroid use: a pilot study to determine whether corticosteroids prevent the diagnosis of primary central nervous system lymphoma. Ann Neurol.

[22] Roman-Goldstein SM, Goldman DL, Howieson J, Belkin R, Neuwelt EA (1992). MR of primary CNS lymphoma in immunologically normal patients. AJNR Am J Neuroradiol.

[23] Haldorsen IS, Espeland A, Larsson EM (2011). Central nervous system lymphoma: characteristic findings on traditional and advanced imaging. AJNR Am J Neuroradiol.

[24] Koeller KK, Smirniotopoulos JG, Jones RV (1997). Primary central nervous system lymphoma: radiologic-pathologic correlation. Radiographics.

[25] Barajas RF, Politi LS, Anzalone N (2021). Consensus recommendations for MRI and PET imaging of primary central nervous system lymphoma: guideline statement from the International Primary CNS Lymphoma Collaborative Group (IPCG). Neuro Oncol.

[26] Fitzsimmons A, Upchurch K, Batchelor T (2005). Clinical features and diagnosis of primary central nervous system lymphoma. Hematol Oncol Clin North Am.

[27] Önder E, Arıkök AT, Önder S (2015). Corticosteroid pre-treated primary CNS lymphoma: a detailed analysis of stereotactic biopsy findings and consideration of interobserver variability. Int J Clin Exp Pathol.

[28] Nguyen-Them L, Costopoulos M, Tanguy ML (2016). The CSF IL-10 concentration is an effective diagnostic marker in immunocompetent primary CNS lymphoma and a potential prognostic biomarker in treatment-responsive patients. Eur J Cancer.

[29] Sasagawa Y, Akai T, Tachibana O, Iizuka H (2015). Diagnostic value of interleukin-10 in cerebrospinal fluid for diffuse large B-cell lymphoma of the central nervous system. J Neurooncol.

[30] Mabray MC, Barajas RF, Villanueva-Meyer JE, Zhang CA, Valles FE, Rubenstein JL, Cha S (2016). The combined performance of ADC, CSF CXC chemokine ligand 13, and CSF interleukin 10 in the diagnosis of central nervous system lymphoma. AJNR Am J Neuroradiol.

[31] Song Y, Zhang W, Zhang L (2016). Cerebrospinal fluid IL-10 and IL-10/IL-6 as accurate diagnostic biomarkers for primary central nervous system large B-cell lymphoma. Sci Rep.

[32] Shao J, Chen K, Li Q (2020). High level of IL-10 in cerebrospinal fluid is specific for diagnosis of primary central nervous system lymphoma. Cancer Manag Res.

